# Impact of diurnal unsymmetrical warming on soil respiration in an agroecological system of the Lhasa region

**DOI:** 10.1371/journal.pone.0217575

**Published:** 2019-05-29

**Authors:** Zhiming Zhong, Guangyu Zhang, Haorui Zhang

**Affiliations:** 1 Lhasa Plateau Ecosystem Research Station, Key Laboratory of Ecosystem Network Observation and Modeling, Institute of Geographic Sciences and Natural Resources Research, Chinese Academy of Sciences, Beijing, China; 2 University of Chinese Academy of Sciences, Beijing, China; Tennessee State University, UNITED STATES

## Abstract

**Purpose:**

The impact of diurnal unsymmetrical rise in temperature on soil respiration (*R*_s_) is not fully understood; thus, we explored such a warming influence on *R*_s_ in an agroecological system of the Lhasa.

**Materials and methods:**

A field warming experiment (C: control; DW: daytime warming; NW: nighttime warming; DW+NW: daytime plus nighttime warming) was carried out in a naked barley ecological system.

**Results and discussion:**

The DW, NW and DW+NW treatments dramatically increased soil temperature and decreased soil moisture but did not markedly modify *R*_s_. The effects of DW and NW on soil respiration sensitivity (*Q*_10_) during the daytime and nighttime were different; they had no effects on daytime *Q*_10_ of *R*_s_, but a significant inhibitory effect on nighttime *Q*_10_ of *R*_s_.

**Conclusions:**

A diurnal unsymmetrical rise in temperature brought about different results for the *Q*_10_ of *R*_s_ but did not cause changes in *R*_s_ under different experimental treatments in agroecological systems of the Lhasa.

## Introduction

Global surface temperature shows a slower increase during the day than at night[1]; due to the complexity of geographic elements and the interaction of climatic factors, there is still a significant asymmetry in global warming[2]. The magnitude of nighttime temperature increase is significantly higher than daytime[3], and both temperatures’ impacts on terrestrial ecological systems are most likely different from each other[4, 5]. For instance, a daytime rise in temperature has a stronger effect on net nitrogen mineralization than a nighttime rise does at the global scale [6]. The Qinghai-Xizang Plateau is experiencing noticeable diurnal unsymmetrical rise in temperature [7]. Although increasing numbers of warming studies have discovered the influence of rise in temperature on cold and high-altitude ecological systems on the Qinghai-Xizang Plateau[8–10], only a few have explored the influence of diurnal unsymmetrical rise in temperature on cold and high-altitude meadows[11, 12]. However, besides these meadows, there are several other cold and high-altitude ecological systems (e.g., agroecological system)[13–20] which have not been researched. Agroecological systems, mainly distributed in semiarid valley districts, are experiencing noteworthy rises in temperature[21–23] and are extremely fragile because of high elevation (generally 3000–4500 m), low air temperature and arid/semiarid climatic circumstances in the Qinghai-Xizang Plateau[24–26]. Therefore, it is necessary to explore how diurnal unsymmetrical warming can affect the agroecological system.

Soil respiration is closely related to the global carbon cycle[27]. This nonuniformity of day-night temperature increase profoundly affects the response and feedback of terrestrial ecosystems’ carbon cycles to global climate change. *R*_s_ is estimated to be more susceptible to rise in temperature in agroecological systems than other ecological systems (e.g., grassland ecological systems) considering that rural soils have better soil moisture (SM) and ventilation [28]. Several studies have explored the impact of experimental warming on *R*_s_ in a variety of agroecological systems [29, 30]; but there are inconsistent results, reporting both positive[11] and negligible[31, 32] effects, on the impact of warming on *R*_s_ in the Qinghai-Xizang Plateau. Although increasing numbers of studies concentrate on the impact of experimental warming on *R*_s_ in forest and grassland ecological systems[8, 32], only two studies have explored the influence of rise in temperature on *R*_s_ in agroecological systems of the Qinghai-Xizang Plateau [29, 30]. These two studies only explored the influence of rise in temperature on *R*_s_ during a single growing season, whereas the impact of rise in temperature on *R*_s_ may change within the year in cold and high-altitude districts [33]. Furthermore, [30] reports the impact of DW+NW treatment on *R*_s_ only during the daytime (8:00–20:00), while [29] shows the impact of DW+NW treatment on *R*_s_ during only the daytime (8:00–20:00) and only nighttime (20:00–8:00). Therefore, how diurnal unsymmetrical warming can modify *R*_s_ in agroecological systems of the Qinghai-Xizang Plateau remains unclear.

To the best of our knowledge, studies of the effect of diurnal unsymmetrical warming on *R*_s_ in the Qinghai-Tibet Plateau have only been carried out in alpine meadows[11]. However, the response of *R*_s_ to asymmetric warming in agroecological systems has not been studied. Since the naked barley ecological system is one of the dominant agroecological systems in the Lhasa[34], a diurnal unsymmetrical warming experiment (C: control; DW: 8:00–20:00; NW: 20:00–8:00; DW+NW: 8:00–8:00) was carried out during two growing seasons in 2015–2016 to compare the impacts of the DW, NW and DW+NW treatments on *R*_s_ and *Q*_*10*_ of *R*_s_ in such an ecological system of the Lhasa region.

## Materials and methods

### Research area and experimental design

The study area is located at the Lhasa Agroecosystem Research Station, Tibet Autonomous Region in China(91°21′E, 29°41′N, 3688 m). The annual average temperature is 7.9°C, and the average annual precipitation is 425 mm. Since the 1970s, the soil in this area has been used for cultivating crops. A number of studies described the research area conditions (e.g., climate traits) [29, 30]. Infrared heaters (Kalglo Electronics Inc., Bethlehem, PA, USA) were positioned on the ground approximately 1.7 m in the center of each 2 m×2 m plot and used to enhance soil temperature (*T*_s_) during the two growing seasons of a naked barley ecological system (April 23–August 24 in 2015; April 15–August 16 in 2016). The dimensions and installation heights of infrared heaters, sowing rates and rows of naked barley, and sizes of and distances between sample plots matched those given in a previous study [29]. The field experiment was completely randomized design, with four treatments(C, DW, NW and DW+NW) and four replications per treatment. Both the Lhasa Agroecosystem Research Station and the village leaders gave us permission to conduct our study on their land. All the plants we used were planted by the experimental staff on the station.

### Biomass, microclimate and *R*_*s*_ measurements

We sampled aboveground and belowground portions of naked barley on August 16, 2016, and rinsed any residual soil off with water. Then, the plant sample was dried at a constant temperature of 65°C for 48 h; the weight of the aboveground vegetation was the aboveground biomass, and the weight of the root was the underground biomass.

We measured soil moisture (SM) and temperature (*T*_s_) (5 cm) during the whole research period in 2015–2016. In the center of each plot, we set *T*_s_ and SM sensors at a depth of 0.05 m. For each plot, the data of the two sensors was exported via the data logger (HOBO weather station, Onset Computer, Bourne, MA, USA). Data were measured every minute and processed to provide an average value every 5 minutes.

We used LI-8100 (LI-COR Biosciences, Lincoln, NE, USA) to measure daily cycles of *R*_s_ during the entire research period in 2015–2016. The *R*_s_ observation interval was 2 h during the daytime (8:00 to 20:00) and 3 h and during the nighttime (20:00 to 8:00). A previous study [29] has shown the sample setting of the *R*_s_ measuring equipment.

### Statistical analyses

We evaluated the impacts of warming and the observation date on *R*_s_ using repeated-measures ANOVA. We used an independent sample t-test to compare the seasonal mean soil respiration for the two growing seasons under the four treatments. We explored exponential correlations between *R*_s_ and *T*_s_ using the Eq 1 and *Q*_10_ of *R*_s_ using the Eq 2 according to previous studies [29, 31].

$$R_{s}=ae^{bT_{s}},$$ (1)

$$Q_{10}=e^{10b}$$ (2)

We performed natural-logarithm transformations between *R*_*s*_ and SM and then analyzed the correlations between *R*_s_, *T*_s_ and SM using a multiple stepwise regression analysis. We calculated *R*_s_ and *Q*_10_ for the full day (8:00–8:00), day (8:00–20:00), and night (20:00–8:00), and compared *R*_s_ and *Q*_10_ of three periods among the 'C','DW', 'NW' and 'DW+NW' treatments with Student-Newman-Keuls multiple comparisons.

All statistical analyses were performed in the SPSS software (version 22.0; SPSS Inc., Chicago, IL, USA).

## Results

### Impacts of DW, NW and DW+NW treatments on *T*_*s*_, SM and the correlations between *R*_*s*_ and *T*_*s*_

**Fig 1:**
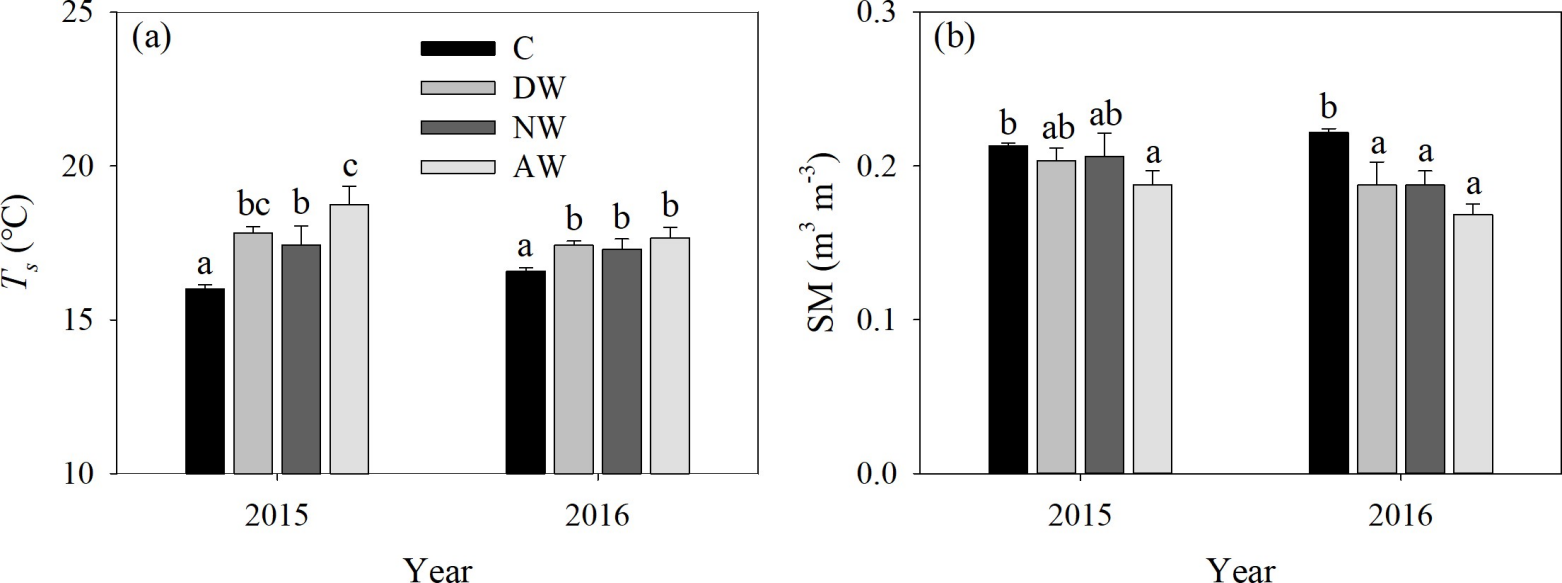
Comparison of soil temperature and soil moisture among diverse warming treatments. (a) is soil temperature (*T*_s_) and (b) is soil moisture (SM) among diverse warming treatments in 2015 and 2016. C: control; DW: daytime warming; NW: nighttime warming; DW+NW: daytime plus nighttime warming.

**Fig 2:**
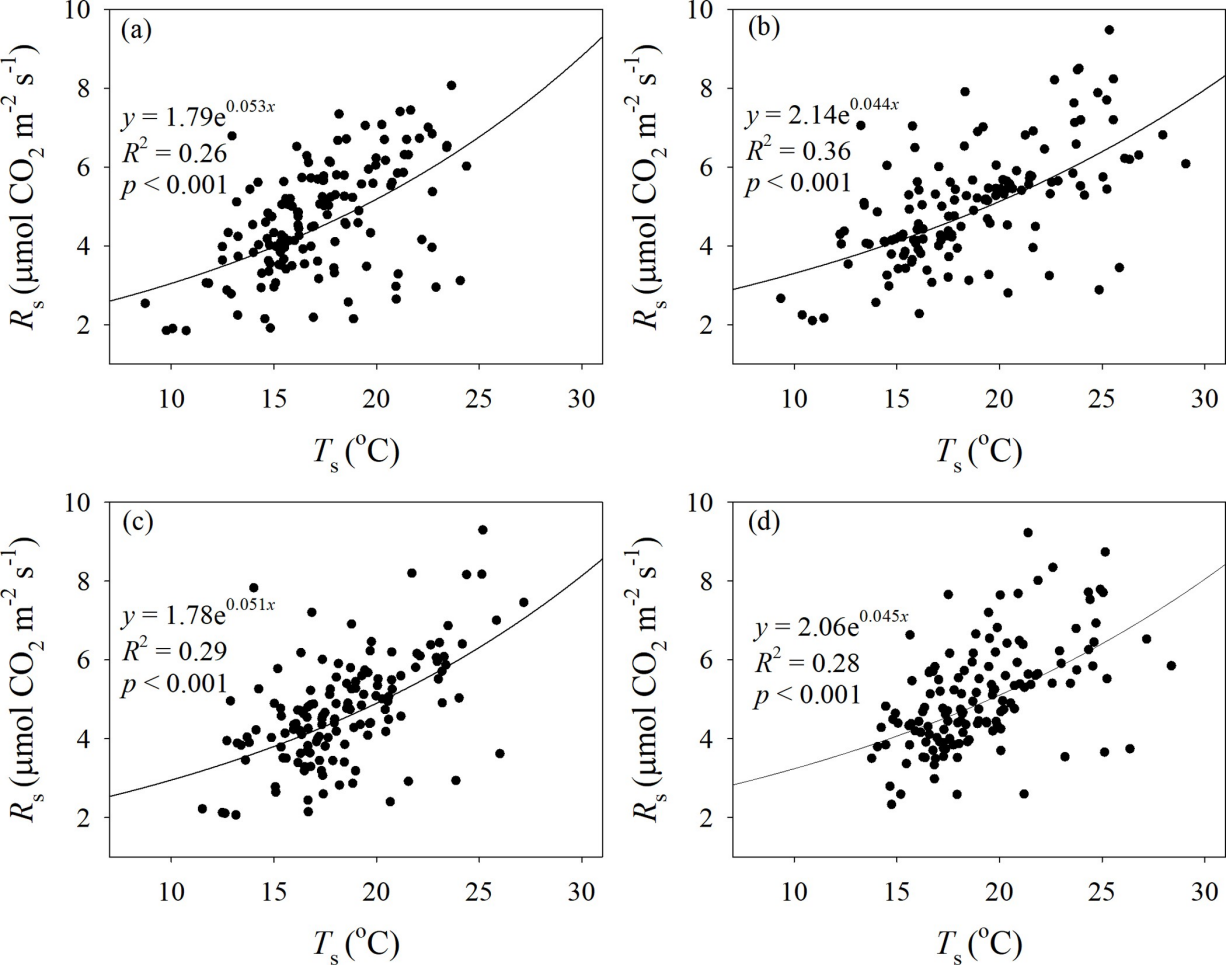
Relationships between *R*_s_ and *T*_s_. *R*_s_: soil respiration. *T*_s_: soil temperature.(a) control, (b) daytime warming, (c) nighttime warming and (d) daytime plus nighttime warming.

The comparisons of *T*_s_, SM and the correlations between *R*_s_ and *T*_s_ among the C, DW, NW and DW+NW treatments are illustrated in Figs 1 and 2. The DW, NW and DW+NW treatments dramatically raised *T*_s_ by 1.33, 1.07 and 1.91°C in 2015–2016; by 1.83, 1.44 and 2.74°C in 2015; and by 0.84, 0.71 and 1.08°C in 2016, all respectively. The DW, NW and DW+NW treatments dramatically decreased SM by 0.02, 0.02 and 0.04 m^3^ m^-3^ in 2015–2016 and by 0.03, 0.03 and 0.05 m^3^ m^-3^ in 2016, respectively. The DW+NW treatment dramatically decreased SM by 0.02 m^3^ m^-3^ in 2015, while the DW and NW treatments did not dramatically modify SM. *R*_s_ increased exponentially with increasing *T*_s_ (Fig 2). The *p*-values of all equations are significant (*p*<0.001); the exponential equation provides a good fit for the relationship between temperature and soil respiration.

### Impacts of DW, NW and DW+NW treatments on *R*_*s*_, temperature sensibility and plant biomass

**Fig 3:**
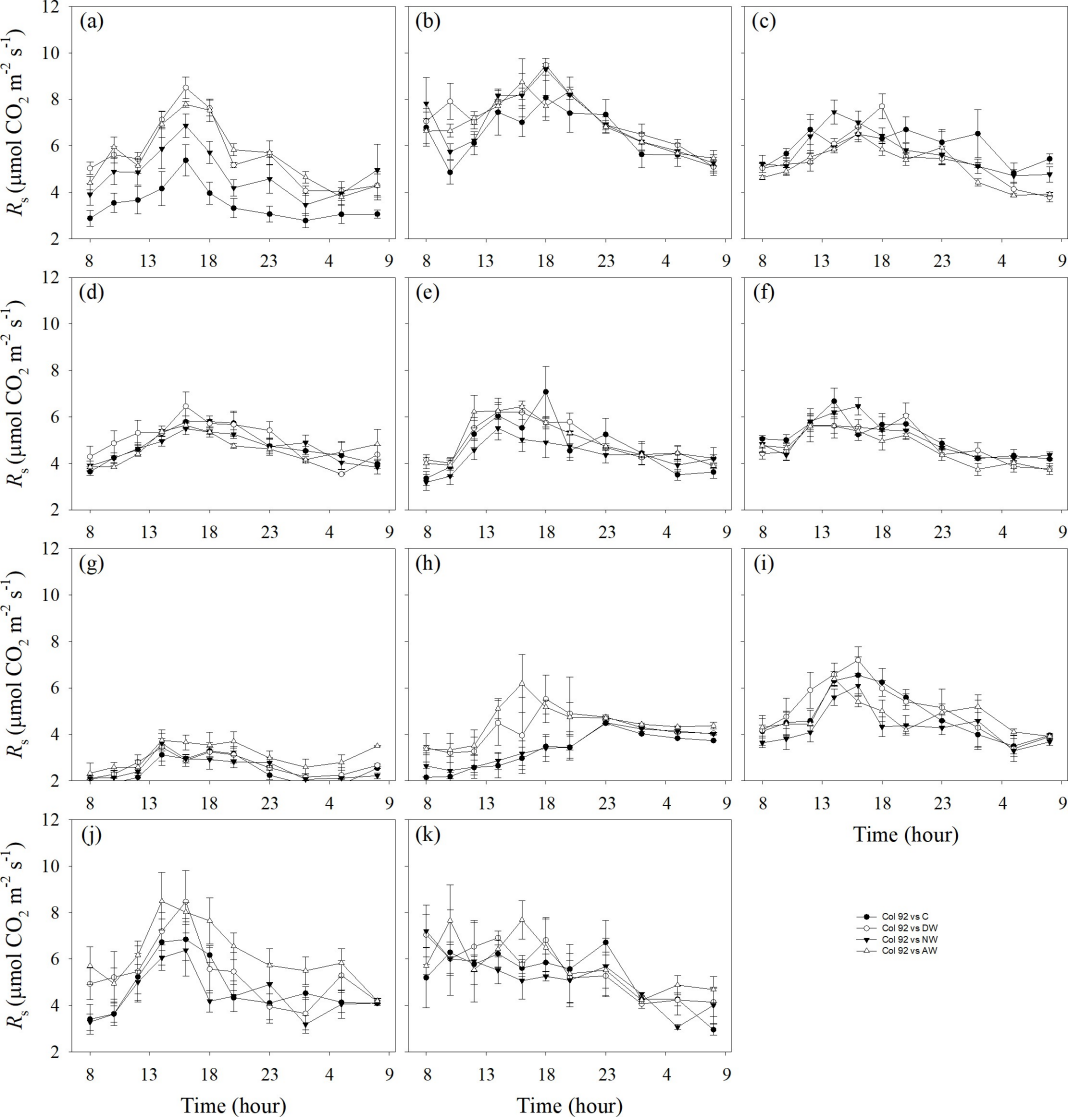
Comparison of *R*_s_ among diverse warming treatments in different months. *R*_s_: soil respiration (a) May 25–26, 2015, (b) June 16–17, 2015, (c) June 30–July 1, 2015, (d) July 16–17, 2015 (e) July 30–31, 2015, (f) August 14–15, 2015, (g) May 6–7, 2016, (h) June 12–13, 2016, (i) July 16–17, 2016, (j) July 27–28, 2016 and (k) August 10–11, 2016. C: control; DW: daytime warming; NW: nighttime warming; DW+NW: daytime plus nighttime warming.

**Fig 4:**
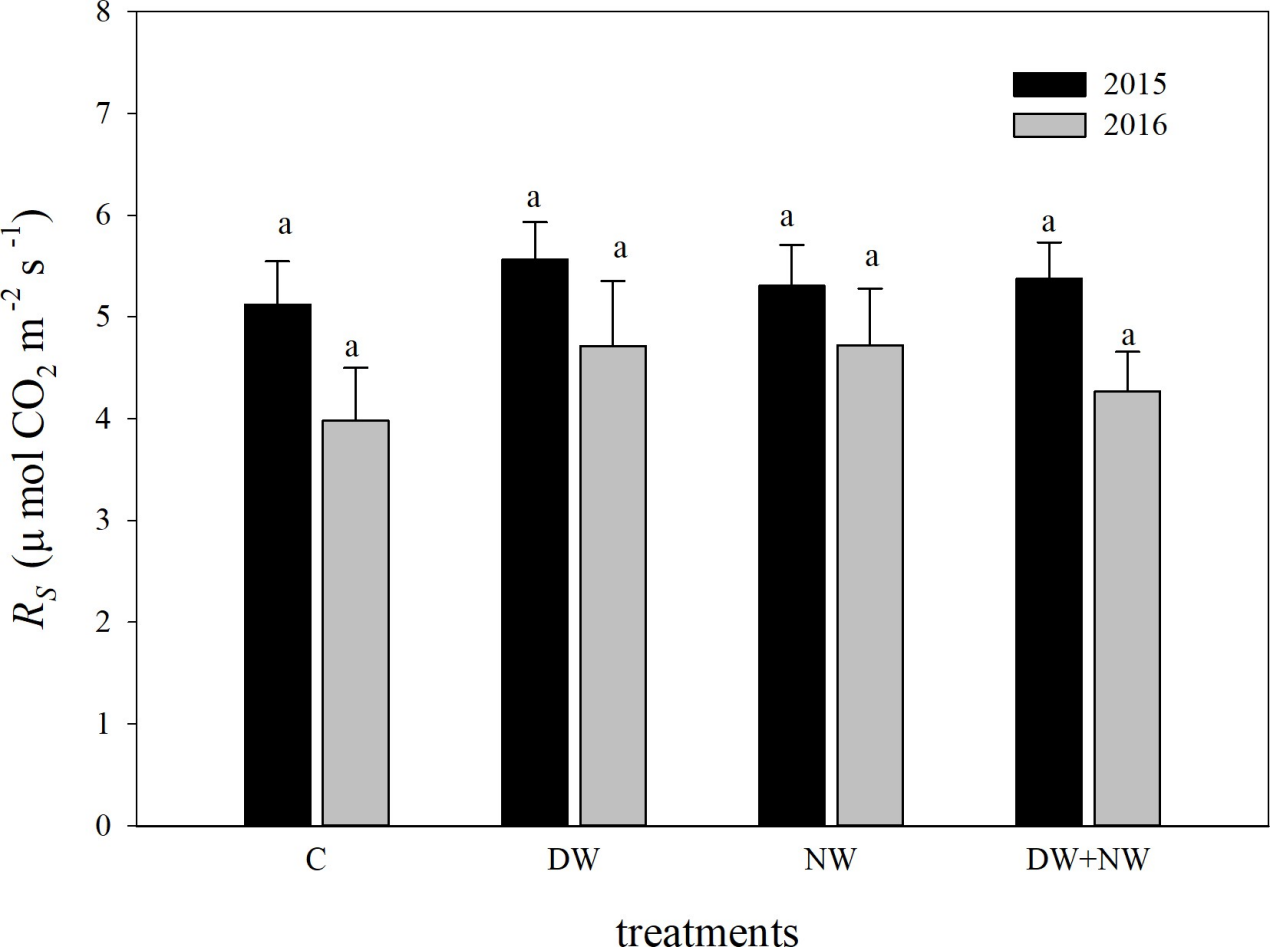
Comparison of the mean *R*_s_ for the two growing seasons under the four treatments. *R*_s_: soil respiration. C: control; DW: daytime warming; NW: nighttime warming; DW+NW: daytime plus nighttime warming.

The main impact of the observation date and its interaction impact with warming on *R*_s_ of the all-day treatment were remarkable. While the main impact of warming on *R*_s_ was insignificant, there was a significant difference in soil respiration intensity between different stages of the growing seasons in 2015 and 2016 (Table 1). Through Student-Newman-Keuls multiple comparison, we found *R*_s_ of all-day treatment among the C, DW, NW and DW+NW treatments had no significant difference. The comparisons of *R*_s_ among the C, DW, NW and DW+NW treatments are illustrated in Fig 3, while the comparison of the seasonal mean soil respiration for the two growing seasons under the four treatments is illustrated in Fig 4. Through an independent sample t-test, it was found that there was no significant difference in seasonal mean soil respiration between the two years.

**Table 1:** Repeated-measures ANOVA was used to estimate the main and interaction impacts of warming (W) and observation date (D) on soil respiration (*R*_s_) during the growing seasons in 2015 and 2016.

Year	Model	*F*	*p*
2015	W	0.79	0.525
	D	42.93	<0.001
	W×D	3.58	<0.001
2016	W	1.67	0.226
	D	93.75	<0.001
	W×D	2.54	0.036

**Fig 5:**
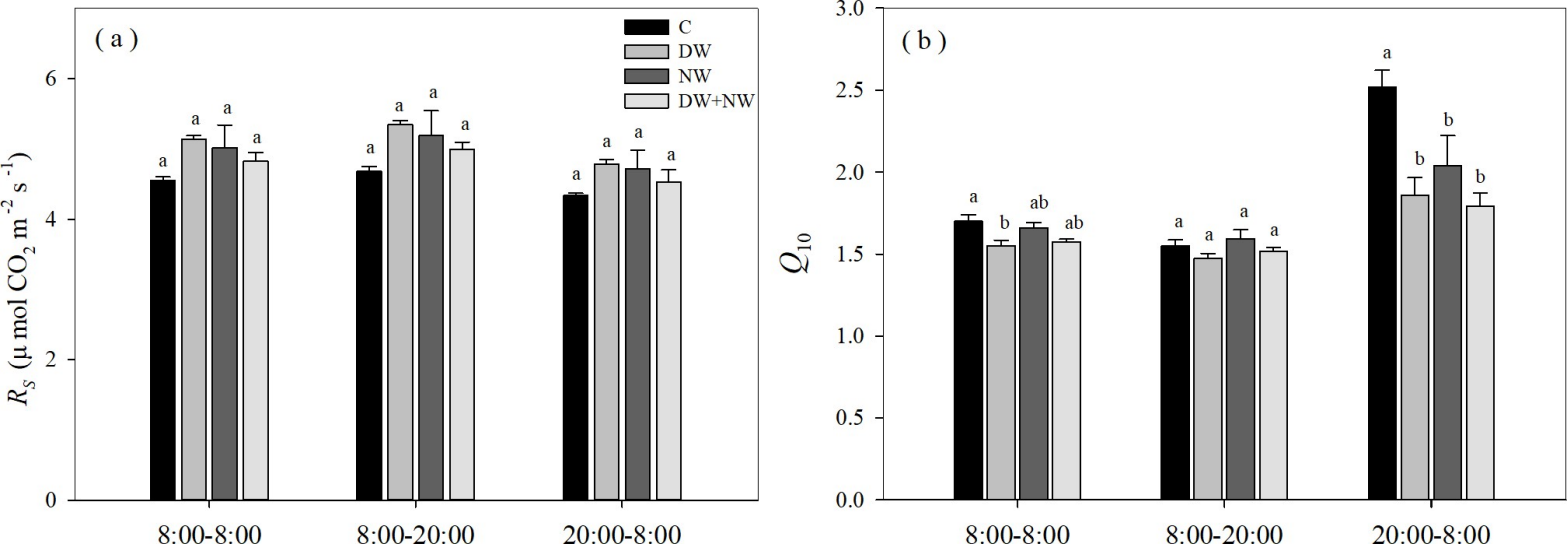
Comparison of four treatments in the *R*_*s*_ *and Q*_10_ of three time periods. *R*_s_: soil respiration .*Q*_10_: temperature sensibility of soil respiration. The four treatments were C: control; DW: daytime warming; NW: nighttime warming; and DW+NW: daytime plus nighttime warming. The three time periods were 8:00–8:00, 8:00–20:00, and 20:00–8:00.

**Fig 6:**
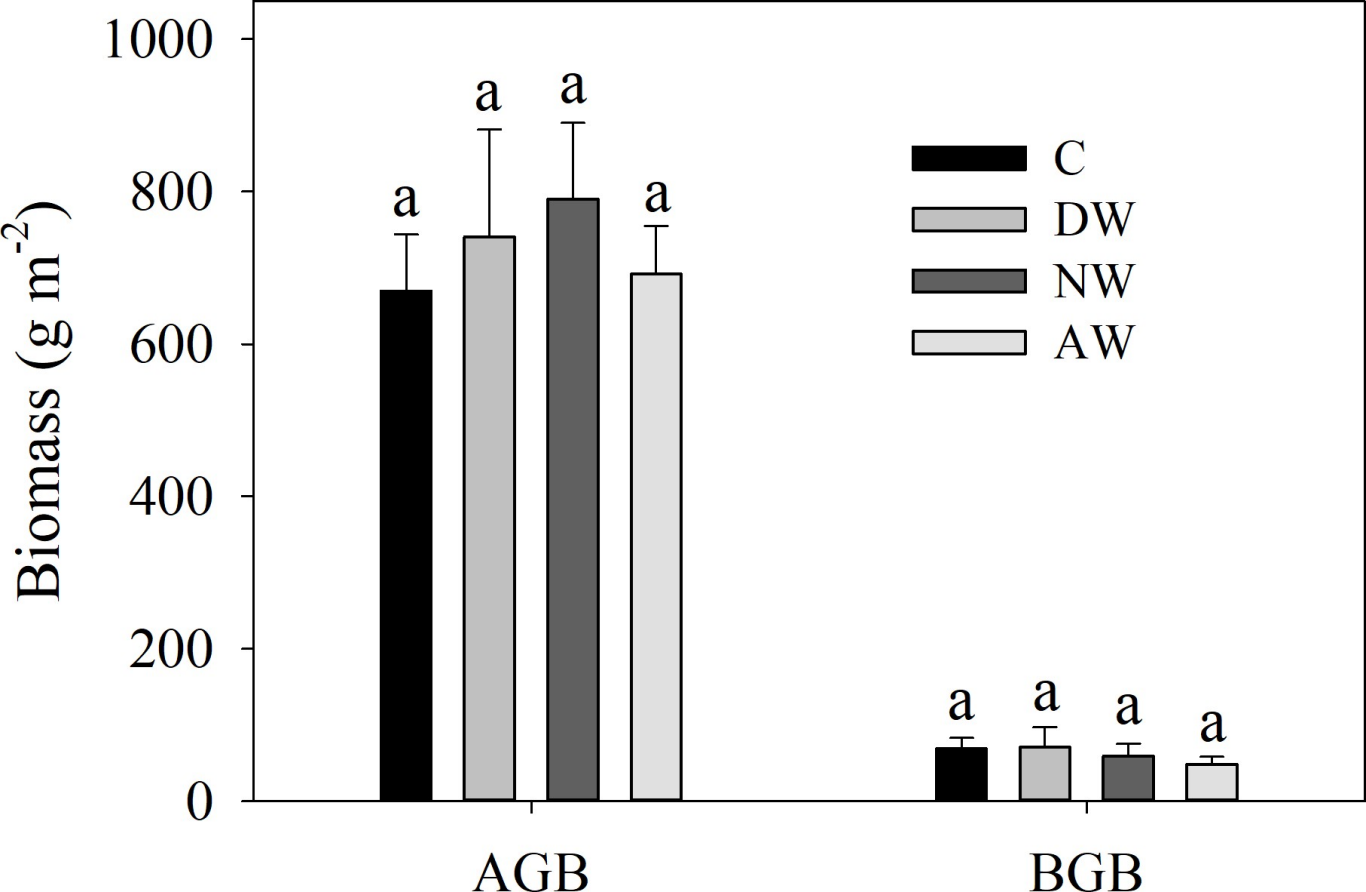
Comparison of AGB and BGB among diverse warming treatments in 2016. AGB: aboveground biomass; BGB: belowground biomass. C: control; DW: daytime warming; NW: nighttime warming; DW+NW: daytime plus nighttime warming.

The effects of the four treatments (C, DW, NW and DW+NW) on daytime and nighttime soil respiration showed no significant difference (Fig 5A). The effects of four treatments (C, DW, NW and DW+NW) on the daytime and nighttime *Q*_10_ were different, while those on the daytime *Q*_10_ had no significantly difference; Compared with the C treatment, DW, NW and DW+NW had significant inhibitory effects on the nighttime *Q*_10_, with all treatments having no significant difference between them. From the all-day *Q*_10_, only C and DW showed a significant difference (Fig 5B). From Fig 2, the b values of Eq 1 can be used to calculate the *Q*_10_ value. The b values obtained by fitting the four treatments were 0.053, 0.044, 0.051, 0.045. The difference between the C and DW treatments was the largest, consistent with the multiple comparison results of all-day *Q*_10_ between the four treatments (Fig 5B). The DW, NW and DW+NW treatments had negligible impacts on plant biomass (Fig 6).

### Correlations between R_s_ and temperature sensibility, and T_s_, and SM

**Fig 7:**
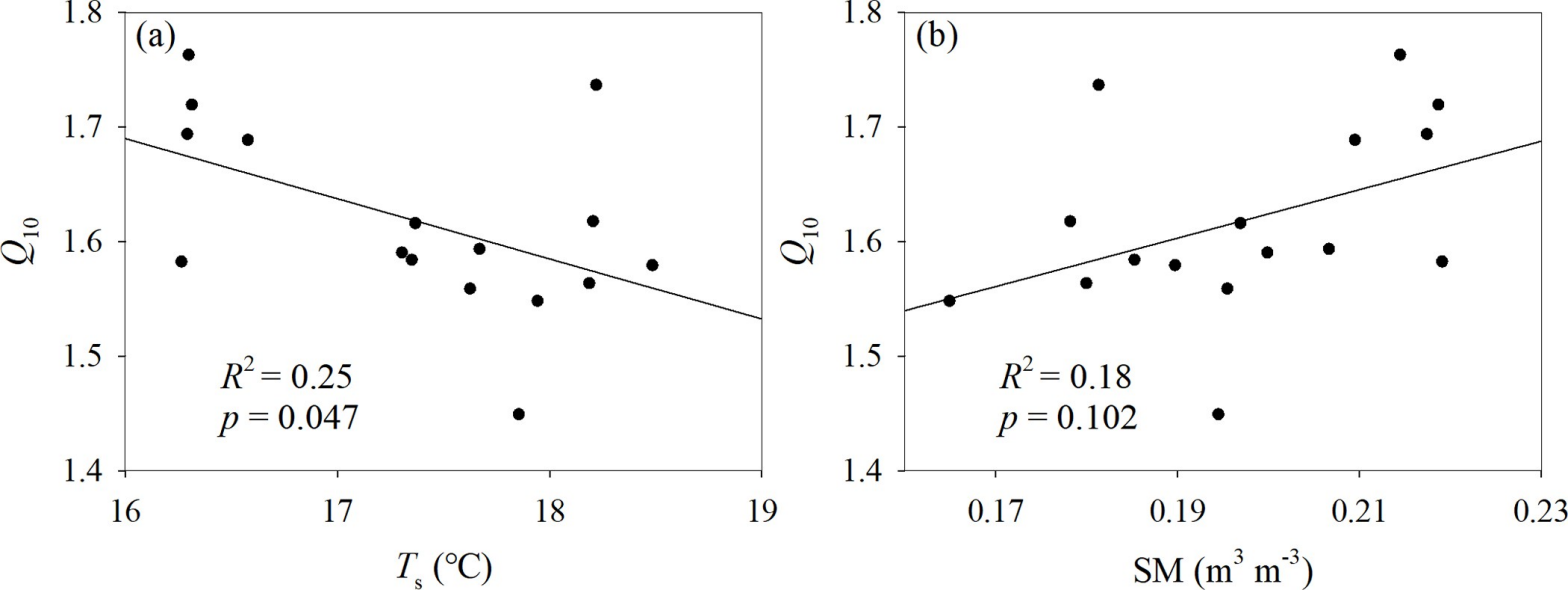
Relationships between *Q*_10_ and *T*_s_ and relationships between *Q*_10_ and SM. (a): Relationships between the temperature sensibility (*Q*_10_) of soil respiration and soil temperature (*T*_s_) (b): Relationships between the temperature sensibility (*Q*_10_) of soil respiration and soil moisture (SM).

The correlations between *R*_s_, *T*_s_ and SM are shown in Table 2. All *p*-values were significant (*p*<0.001) based on different interpretation variables for the equation under different treatments. After the stepwise regression, SM did not enter the equation and *T*_s_ explicated the change of *R*_s_ under the C treatment, whereas *T*_s_ and SM jointly explained the change in *R*_s_ under the DW, NW and DW+NW treatments. The regression equations are: y = 0.05x_1_+0.59, y = 0.04x_1_+0.31x_2_+1.26, y = 0.05x_1_+0.37x_2_+1.18, y = 0.05x_1_+0.16x_2_+0.98 (x_1_ is *T*_*s*_, x_2_ is SM, y is *R*_s_). Partial correlation coefficients of *T*_s_ with respect to the C, DW, NW and DW+NW treatments were 0.05, 0.62, 0.59, and 0.54, respectively; those of the SM with respect to the DW, NW and DW+NW treatments were 0.09, 0.19, and 0.05, respectively; and those between *R*_s_ and *T*_s_ exceeded those between *R*_s_ and SM of the DW, NW and DW+NW treatments. Compared with SM, *T*_s_ can better explain the change in *R*_s_. The *Q*_10_ value decreased significantly with increasing *T*_s_ across all the experimental plots, while the *Q*_10_ value only tended to increase with increasing SM (Fig 7).

**Table 2:** Multiple stepwise regressions between soil respiration (*R*_s_) and soil temperature (*T*_s_) and moisture (SM).

Treatment		Regression coefficient	*R*^2^	Partial correlation coefficient	*p*
C	Constant	0.59			<0.001
	*T*_*s*_	0.05	0.25	0.50	<0.001
DW	Constant	1.26			<0.001
	*T*_*s*_	0.04	0.35	0.62	<0.001
	SM	0.31	0.09	0.36	<0.001
NW	Constant	1.18			<0.001
	*T*_*s*_	0.05	0.28	0.59	<0.001
	SM	0.37	0.19	0.51	<0.001
DW+NW	Constant	0.98			<0.001
	*T*_*s*_	0.05	0.27	0.54	<0.001
	SM	0.16	0.05	0.25	0.003

C: control; DW: daytime warming; NW: nighttime warming; DW+NW: daytime plus nighttime warming.

## Discussion

We have discovered that the degree of soil drought produced by the DW+NW treatment was substantially larger than that by the DW and NW treatments, which possibly originated from the fact that the DW+NW treatment brought about a larger increase in *T*_s_ than did the DW and NW treatments[29, 31, 32, 35]. Our findings suggest that diurnal unsymmetrical warming will not always trigger unsymmetrical results on plant biomass in cold and high-altitude districts, which is possibly due to unsuitable increase in *T*_s_, warming-induced decline in SM and an increase in plant respiration [32, 35–40]. Our findings suggest that warming can modify the relative impacts of *T*_s_ and SM on *R*_s_ in cold and high-altitude districts[29, 31, 41].

Vegetation growth and soil microorganism activity are strongly influenced by low temperatures[8, 10, 42, 43], thus rise in temperature is estimated to amplify *R*_s_ in cold and high-altitude districts [8]. However, several previous studies noted that rise in temperature had no effect on *R*_s_ in agroecological systems and cold and high-altitude meadows in the Qinghai-Xizang Plateau [29, 32, 44], a result which was additionally supported by this study. All of these findings entailed that *R*_s_ most likely has no significant response to warming in cold and high-altitude districts. Our findings did not follow the lines of earlier meta-analyses which showed that *R*_s_ increased during the warming treatments [45, 46].

We have discovered that the *Q*_10_ of *R*_s_ between the DW and NW treatments were diverse, and moreover, that DW and NW treatments can lead to differing effects in the daytime and nighttime *Q*_10_. This indicated that *Q*_10_ was not only related to the different ecosystems and the magnitude of temperature increase, but different warming methods as well. The all-day *Q*_10_ values were 1.69, 1.55, 1.66 and 1.58 for the C, DW, NW and DW+NW treatments, respectively. Compared with C treatment, DW and NW *Q*_10_ values decreased by 9% and2%, respectively, but the DW + NW *Q*_10_ decreased by 7% indicating that the impact of DW and NW was not a simple additive relationship. As can be understood from Fig 5, DW significantly reduced all-day Q_10_ values, while NW and DW+NW had no significant change; this further proves that these two processes were not simple additive relationships. We even speculated that DW and NW may have antagonistic effects; as a consequence, the asymmetry of daytime and nighttime rises in temperature will possibly bring about unsymmetrical results for the temperature sensibility of *R*_s_ in cold and high-altitude rural soils.

*Q*_10_ can be used to reflect the relationship with temperature, as its value is closely related to temperature. From Fig 7, it can be seen that *Q*_10_ is negatively correlated with temperature, which is consistent with previous studies[47–49]. Kirschbaum[50] analyzed the results of different studies and found that *Q*_10_ was very high at low temperatures and relatively stable at high temperatures. Luo [51]found that the *Q*_10_ value of the treatment without warming was significantly higher than the *Q*_10_ value of the experimental warming treatment in a study on a cold and high-altitude grassland in North America. Moreover, we found that the *Q*_10_ value of soil respiration during daytime treatment was lower than that of nighttime (Fig 5B), which further confirmed the negative correlation between temperature and *Q*_10_.

The minor differences of *R*_s_ among the C, DW, NW and DW+NW treatments are possibly due to the following reasons. First, there were minor differences of BGB among the C, DW, NW and DW+NW treatments, and *R*_s_ was positively correlated with BGB [52]. Next, although the DW+NW treatment brought about a larger rise in *T*_s_, it triggered more soil drought than did the DW and NW treatments which in turn was able to lessen the impact of rise in temperature on *R*_s_. Third, the warming degrees of the DW, NW and DW+NW treatments possibly did not reach the optimum temperature which could lead to maximum increase in *R*_s_ [32]. Lastly, only the DW treatments, not the NW and DW+NW treatments, dramatically lowered the *Q*_10_ of *R*_s_, which in turn was able to weaken the impact of rise in temperature on *R*_s_ [53].

## Conclusions

This study explored how diurnal unsymmetrical warming was able to modify *R*_s_ and temperature sensibility of *R*_s_ in an agroecological system within the Qinghai-Xizang Plateau during two growing seasons in 2015–2016. The *R*_s_ differences among the C, DW, NW and DW+NW treatments were able to be neglected. The effects of DW, NW and DW+NW treatments on *Q*_10_ of *R*_s_ during daytime and nighttime were different; they both had no effects on daytime soil respiration sensitivity, but significant inhibitory effects on nighttime soil respiration sensitivity. Moreover, we speculate that DW and NW have antagonistic effects on *Q*_10_ of *R*_s_.

Through warming experiments, we found that the soil respiration under the diurnal unsymmetrical warming has different sensitivities while the magnitude of warming caused by global warming differed between day and night. The experiment explores the effects of the asymmetric warming mechanism on soil respiration, providing a theoretical basis for better simulation and prediction of soil respiration so as to better explain the response and feedback of terrestrial ecosystems’ carbon cycle to global climate change.

## Supporting information

S1 Data Four treatments in the soil respiration of three time periods. (XLSX)

S2 Data Four treatments in the *Q*_10_-value of three time periods. (XLSX)

S3 Data Soil temperature among diverse warming treatments. (XLSX)

S4 Data Soil moisture among diverse warming treatments. (XLSX)

S5 Data AGB and BGB among diverse warming treatments in 2016. (XLSX)

S6 Data The mean *R*_*s*_ for the two growing seasons under the four treatments. (XLSX)
